# Monkeypox-Associated Pericarditis: A Maiden Case

**DOI:** 10.7759/cureus.29638

**Published:** 2022-09-26

**Authors:** Tanveer Ahamad Shaik, Diana Voloshyna, Tayseer H Nasr, Ameer Makki, Suma Harsha Kosuru, Maryam H Khan, Naglaa G Ghobriel, Qudsia I Sandhu, Farhan Saleem

**Affiliations:** 1 Cardiovascular Medicine, University of Louisville School of Medicine, Louisville, USA; 2 School of Medicine, University of Michigan, Ann Arbor, USA; 3 Telemetry, Inova Alexandria Hospital, Alexandria, USA; 4 Medicine, Inova Alexandria Hospital, Alexandria, USA; 5 Internal Medicine, Osmania Medical College, Hyderabad, IND; 6 Internal Medicine, Rawalpindi Medical University, Rawalpindi, PAK; 7 Internal Medicine, University of Alexandria Egypt, Alexandria, EGY; 8 Medicine, D.G Khan Medical College, Dera Ghazi Khan, PAK; 9 Orthopaedic Surgery, Lahore General Hospital, Lahore, PAK

**Keywords:** acute cardiac care, viral pericarditis, pericarditis, pericardial diseases, monkeypox virus, monkeypox diagnosis

## Abstract

One of the most prevalent causes of pericarditis has been identified as virus infection. However, very little is known regarding cardiac involvement as a consequence of monkeypox infection. We describe a rare case of pericarditis with mild pericardial effusion in an immunocompetent adult with a one-week history of monkeypox. To the best of our knowledge, not many case reports are available in the existing literature. This might be the among the first few cases of monkeypox associated pericarditis during the current pandemic. The use of nonsteroidal anti-inflammatory medications, and colchicine to manage pericarditis has been the cornerstone of the therapy. Within two weeks, the patient reported improvement in his symptoms and the resolution of the pericardial effusion.

## Introduction

Monkeypox is a reemerging infection endemic to sub-Saharan Africa caused by the monkeypox virus (MPV). It has since spread to different European and American nations [1]. Fever, headache, fatigue, vomiting, painful maculopapular rash, lymphadenopathy, corneal scarring, conjunctivitis, sepsis, bronchopneumonia, and encephalitis are common symptoms of monkeypox [2].

Pericarditis is an inflammatory disorder characterized by pericardial effusion and inflammation of the pericardium resulting from various causes. It is classified as acute pericarditis, chronic pericarditis, and recurrent pericarditis. Pericarditis is characterized by chest pain, fatigue, dyspnea, palpitations, and cardiogenic shock. The diagnostic methods include patient history, physical examination, changes in the electrocardiogram (ECG), erythrocyte sedimentation rate (ESR), white blood cell (WBC) count, C-reactive protein (CRP), and transthoracic echocardiography (TEE). The principal causes of pericardial diseases are infections and systemic diseases. Using polymerase chain reaction (PCR), pericarditis caused by a virus can be diagnosed. It can be treated with nonsteroidal anti-inflammatory drugs, steroidal medications, and colchicine [3]. According to the literature review, viral infections are linked to pericarditis, with coronavirus disease 2019 (COVID-19) and smallpox patients having a serious but uncommon incidence of pericarditis. Our investigation focuses on the viral etiology of pericarditis and its incidence in patients with monkeypox. 

## Case presentation

**Figure 1:**
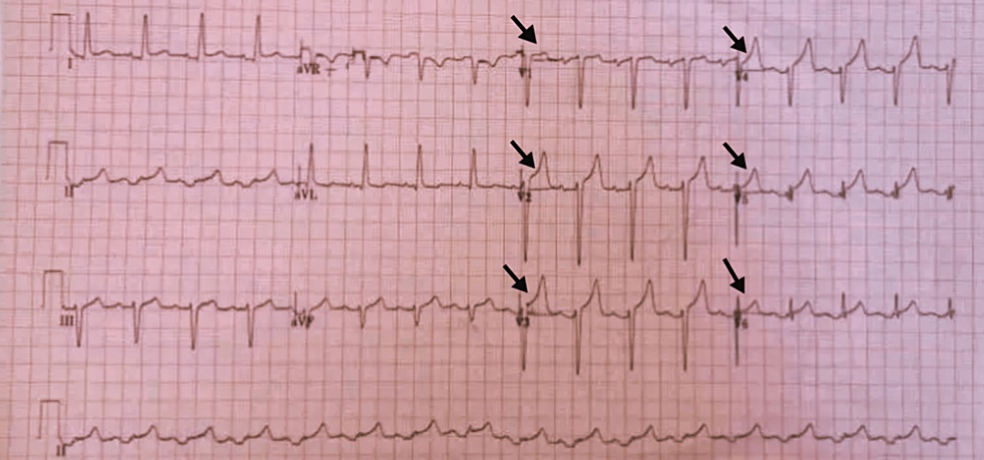
Electrocardiogram (ECG) of the Patient Showing Widespread ST-Elevation.

A 51-year-old Asian man with a one-week history of monkeypox presented to the emergency department with a history of retrosternal chest pain radiating to the left arm. The intensity of the pain was severe, and the patient felt chest tightness after the commencement of the pain. The pain was alleviated by bending forward and exacerbated by swallowing and breathing, especially by inspiration. He had no history of severe illness or any conventional risk factors for heart disease. Seven days prior to presentation, the patient started feeling fatigued with a gradual progression of fever, flu-like symptoms and malaise. The patient self-prescribed paracetamol, but it was ineffective. The patient, after that, developed several vesiculopustular lesions on his face and extremities. PCR confirmed the provisional diagnosis of monkeypox. The patient received supportive care and had been feeling better until the night prior to the presentation, when this episode of chest pain began. An ECG was performed on the spot along with all the basic labs, including two sets of cardiac biomarkers, trop-I and trop-T, six hours apart. His electrocardiogram revealed sinus tachycardia with widespread ST elevation in leads v1-v6 (Figure 1). Nitroglycerin was administered, but it did not alleviate the patient's pain. Laboratory tests are given in Table 1.

**Table 1:** Laboratory Parameters of the Patient. Hb: hemoglobin, MCV: mean corpuscular volume, WBC: white blood cell, ALT: alanine transaminase, AST: aspartate aminotransferase, BUN: blood urea nitrogen, Cr: creatinine, CRP: C-reactive protein, ESR: erythrocyte sedimentation rate

Complete blood count parameters	Value
Hb (g/dL)	13.6 (13.5 - 17.5)
MCV (fl)	76.8 (80 - 100)
WBC (X10^9^/l)	12.2 (4.5 - 11)
Platelets (X10^3^/ul)	230 (150 - 400)
ALT (IU/L)	24 (7 to 55)
AST (IU/L)	33 (8 to 48)
BUN (mg/dL)	24 (6 to 24)
Cr (mg/dL)	1.1 (0.7 to 1.3)
CRP (mg/L)	65.5 (10 or less)
ESR (mm/hr)	34 (1 to 13)
Troponin I (ng/mL)	0.02 (0 to 0.04)
Troponin T (ng/mL)	0 (0 to 0.01)
HbA1c%	5.1 (Below 5.7%)

**Figure 2:**
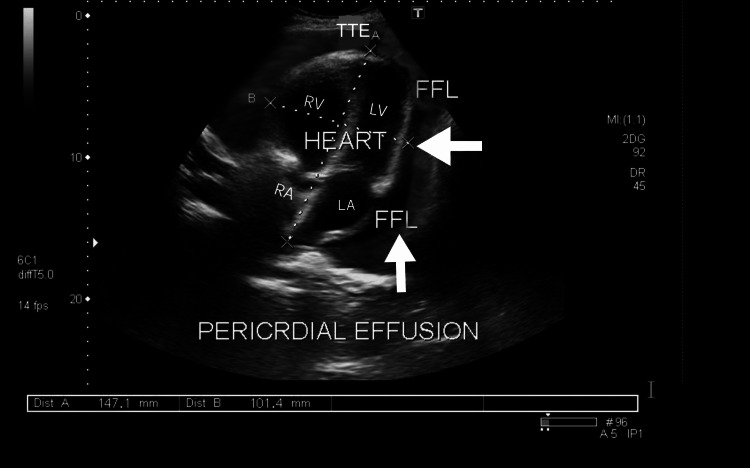
Transthoracic echocardiography (TEE) Subcostal View Showing Pericardial Effusion.

His cardiac biomarkers were found to be normal. His chest x-ray did not show any acute pathology. The patient was found to be diaphoretic and lethargic upon examination. His heart rate was 95 bpm, his blood pressure was 120/80 mmHg, and his temperature was 101 degrees Celsius. A thorough evaluation of the cardiovascular system revealed a friction rub in systole and diastole, with normal S1 and S2. The TTE showed good bi-ventricular function with preserved systolic ejection fraction over 55%, hyperdynamic systolic function, no constrictive processes, no wall motion abnormalities and the presence of mild pericardial effusion (Figure 2).

After ruling out other common causes of pericarditis in the patient, considering the patient's history of monkeypox infection, and identifying three diagnostic criteria for pericardial inflammation, monkeypox-associated pericarditis was determined to be the most likely cause. The patient was immediately admitted to the critical care unit (CCU) and received supportive care. The patient was prescribed 1 gram of aspirin every eight hours for the next two weeks. On the seventh day after admission, the patient's inflammatory markers improved, his CRP was less than 20mg/l, and a repeat TTE revealed no evidence of effusion with a normal left ventricular ejection fraction of > 55%. The patient was discharged on the seventh day with high-dose aspirin, and a planned cardiac follow-up was advised.

## Discussion

Monkeypox is a viral disease prevalent in Central African nations, particularly the Democratic Republic of the Congo [4]. In 1970, the first reported case of monkeypox was documented in Central Africa. It is more prevalent in developing and underdeveloped nations. Monkeypox is caused by the monkeypox virus, which belongs to the genus orthopoxvirus. This genus also contains the variola and cowpox virus, the respective agents responsible for smallpox and cowpox [5]. In May of 2022, Europe was the first region to report a worldwide outbreak of monkeypox. Communities worldwide have been affected by this epidemic, as new cases have been documented in areas where the disease is not endemic. The World Health Organization designated the monkeypox epidemic a worldwide emergency on July 23, 2022. The fact that most instances have been found in men who have sex with men (MSM) suggests that MPV may be transferred through close contact during sexual activity. According to a study of 528 verified cases of human monkeypox infection in 16 countries, 98% of the victims were MSM. Young children have been affected by household transmission [6].

The clinical manifestations of monkeypox consist of a maculopapular rash with lymphadenopathy. The severity of the disease ranges from mild to moderate and severe. Patients report corneal scarring, conjunctivitis, vomiting, bronchopneumonia, sepsis, and encephalitis as additional complications [7]. Recent research indicates that smallpox vaccines are 85% effective in preventing monkeypox infection [8].

Pericarditis is characterized by pericardial inflammation in response to various stimuli that cause an autoimmune or inflammatory response. Pericarditis is the most frequent of the pericardial diseases. It may result in pericardial effusion, which may impair cardiac filling. It is classified as acute, chronic, and recurrent pericarditis [9]. The annual incidence rate of acute pericarditis in an urban region of Italy is 27.7 per 100,000 people. A comparison of prevalence rates based on gender revealed that men aged 16 to 65 were more susceptible to pericarditis than women. It was discovered that young adults are at a greater risk than the general population [10]. After the initial episode of pericarditis, 15-30% of patients experience a recurrence, and approximately 50% of patients experience a second recurrence within 18 months [11]. As most acute episodes of pericarditis are preceded by a flu-like or gastrointestinal syndrome [9,10], the etiological agents behind pericarditis are predominantly viruses. It has long been recognized that tuberculosis is a significant risk factor for the progression of pericarditis and is frequently associated with HIV, particularly in sub-Saharan Africa [10].

Not many cases of cardiac involvement in monkeypox infection have been reported. Vaccines against smallpox were also linked to an increased risk of pericarditis among males [12,13]. Pericarditis and myocarditis are uncommon complications of smallpox vaccination. Myocarditis occurred at a rate of 463 (95% confidence interval [CI] = 150-1079)/100,000 population or 214 (95% CI = 65-558) times the background rate in healthy controls in a prospective study of more than a thousand healthy adults in the United States who received live-attenuated vaccinia vaccine between 2004 and 2010 [14]. Patients with cardiac issues, family history of myocardial infarction, and hypertension should receive the appropriate care. There were 21 cases of pericarditis, myocarditis, or myopericarditis among 37,901 civilian smallpox vaccinees; nine people were hospitalized, but no mortality was reported. This corresponds to an estimated rate of 55 per 100,000 people [15].

The primary therapies to treat pericarditis include colchicine, acetylsalicylic acid, and nonsteroidal anti-inflammatory drugs (NSAIDs) [11]. Patients who were given aspirin (1.2 g) thrice daily for two weeks showed significant improvement. Within eight days, the patient displayed signs of recovery. Randomized controlled trials demonstrate colchicine's efficacy and rapid recovery in pericarditis patients. Patients who do not respond to colchicine and NSAIDs receive low-dose steroids as a second line of treatment [11]. 

Cardiac involvement could be one of the more unusual symptoms of monkeypox. This case report highlights the emergence of monkeypox infection as a primary cause of viral pericarditis with mild pericardial effusion. Physicians should be aware of this unusual relationship and respond appropriately by performing additional diagnostic tests to establish a definitive diagnosis. Pericarditis, whether from an early or late stage of monkeypox infection, should be considered in a patient with chest pain and ST-segment elevation during the course of monkeypox infection. 

## Conclusions

Cardiac involvement might be one of the unusual manifestations of monkeypox infection. This case demonstrates the need to maintain a high index of suspicion for the link between pericarditis and monkeypox when a patient appears with clinical symptoms and a characteristic ECG in the context of monkeypox. It may aid in gaining a new perspective on this burgeoning pandemic and in advancing public health authorities' understanding of such complications. 
